# Improved detection of artifactual viral minority variants in high-throughput sequencing data

**DOI:** 10.3389/fmicb.2014.00804

**Published:** 2015-01-22

**Authors:** Matthijs R. A. Welkers, Marcel Jonges, Rienk E. Jeeninga, Marion P. G. Koopmans, Menno D. de Jong

**Affiliations:** ^1^Department of Medical Microbiology, Academic Medical CentreAmsterdam, Netherlands; ^2^Centre for Infectious Disease Control, National Institute for Public Health and the EnvironmentBilthoven, Netherlands; ^3^Department of Viroscience, Erasmus Medical CenterRotterdam, Netherlands

**Keywords:** high-throughput sequencing, minority variants, Illumina HiSeq2000, influenza virus

## Abstract

High-throughput sequencing (HTS) of viral samples provides important information on the presence of viral minority variants. However, detection and accurate quantification is limited by the capacity to distinguish biological from artificial variation. In this study, errors related to the Illumina HiSeq2000 library generation and HTS process were investigated by determining minority variant frequencies in an influenza A/WSN/1933(H1N1) virus reverse-genetics plasmid pool. Errors related to amplification and sequencing were determined using the same plasmid pool, by generation of infectious virus using reverse genetics followed by in duplo reverse-transcriptase PCR (RT-PCR) amplification and HTS in the same sequence run. Results showed that after “best practice” quality control (QC), within the plasmid pool, one minority variant with a frequency >0.5% was identified, while 84 and 139 were identified in the RT-PCR amplified samples, indicating RT-PCR amplification artificially increased variation. Detailed analysis showed that artifactual minority variants could be identified by two major technical characteristics: their predominant presence in a single read orientation and uneven distribution of mismatches over the length of the reads. We demonstrate that by addition of two QC steps 95% of the artifactual minority variants could be identified. When our analysis approach was applied to three clinical samples 68% of the initially identified minority variants were identified as artifacts. Our study clearly demonstrated that, without additional QC steps, overestimation of viral minority variants is very likely to occur, mainly as a consequence of the required RT-PCR amplification step. The improved ability to detect and correct for artifactual minority variants, increases data resolution and could aid both past and future studies incorporating HTS. The source code has been made available through Sourceforge (https://sourceforge.net/projects/mva-ngs).

## Introduction

RNA viruses are present within hosts as a cloud of closely related non-identical genomes, often referred to as the viral quasispecies (Lauring and Andino, 2010; Domingo et al., 2012). The development of high-throughput sequencing (HTS) technologies has provided the opportunity to investigate the viral quasispecies composition and detection of viral minority variants at frequencies well below 10–20%, generally accepted as the detection limit of Sanger population sequencing (Leitner et al., 1993; Palmer et al., 2005). However, it is essential to distinguish true biological variation from artificial variation introduced by the laboratory and sequencing methods used. Over time, several approaches to error-correction have been developed incorporating complicated probabilistic methods. These methods use the read sequence information but ignore the corresponding phred quality scores (Zagordi et al., 2011; Beerenwinkel et al., 2012; Yang et al., 2013). In addition, most error-correction algorithms were designed for invariable genomes like the human genome, and generally assumed that errors were not only random and infrequent, but can be corrected using the majority of the reads that have the correct base (Beerenwinkel et al., 2012; Yang et al., 2013). For usage in viral population sequencing this assumption does not apply as each variant in theory could represent a unique viral variant, and different approaches for viral quasispecies reconstruction have been designed, like local haplotype reconstruction incorporated in Shorah (Zagordi et al., 2010a,b, 2011). However, these methods still rely solely on read sequence information requiring prior QC steps. In addition, while Shorah performs well using long read sequences and relatively small sized datasets, typical for Roche 454 data, it does not scale well to the shorter reads and much larger Illumina datasets as it can only handle up to one million reads, limiting the usability with Illumina sequencers.

As different HTS platforms utilize different sequencing methods we showed in a previous study on serially passaged influenza A(H1N1)pdm09 virus, that while identical minority variants were detected using the Roche 454 and Illumina GAIIx HTS platforms, the frequencies of these variants could vary depending on the platform used (Watson et al., 2013). Sequencing chemistries of these HTS platforms have been upgraded to produce longer read lengths with improved error profiles which might lead to more sensitive and reliable detection of viral minority variants (Minoche et al., 2011).

The aim of the current study was to identify and characterize the type of technical errors that were introduced due to the RT-PCR amplification and HTS process itself. Therefore, HTS datasets were generated directly from a pool of eight influenza A virus reverse genetics plasmids as well as from RT-PCR amplified viral RNA produced by reverse genetics using the same plasmid pool. After current “best practice” QC, mismatch frequencies per reference genome position were reduced in all three samples, but a large number of minority variants were still identified and assumed to be the result of the RT-PCR amplification and sequencing procedures. We analyzed these minority variants in more detail and show that by using two additional bioinformatic analysis steps, applicable to large datasets, 95% of erroneous bases were detected and could be corrected for. Additionally, we then demonstrate the usability of our approach using three influenza A positive clinical samples. Finally, we discuss the flexibility of our methods for the application to HTS datasets generated from other platforms.

## Materials and methods

### Plasmid DNA and reverse genetics

Reverse genetic system plasmids containing the individual full-length segments of influenza A/WSN/33(H1N1) virus PB2, PB1, PA, HA, NA, NP, MP, and NS in a pHW2000 backbone were kindly provided by Webster. Plasmid DNA was isolated using Maxi prep column purification according to the manufacturer's protocol (Macherey-Nagel). An 80% confluent monolayer of epithelial human embryonic kidney (HEK) 293T/17 cells (ATCC no. CRL-11268) in a T25 tissue culture flask was transfected with an equimolar mixture of the eight WSN33 reverse genetic plasmids (1 μg DNA each) and 20 μl Lipofectamine 2000 (Invitrogen) according to the manufacturer's protocol. Medium was replaced 16 h after transfection with Dulbecco's modified eagle medium (DMEM; Invitrogen) containing 1% fetal calf serum (FCS). Virus-containing supernatant was harvested 48 h after transfection, and cells and cell debris were removed by centrifugation [4 min at 400 relative centrifugation force (RCF)] and filtration through a 0.2-μm filter (FP 030/3; Schleicher and Schuell). For RNA extraction and consecutive full-genome amplification, 200 μl supernatant was added to 400 μl lysis binding buffer (High Pure RNA isolation kit; Roche). The remaining supernatant was aliquoted and stored at −80°C.

### Clinical samples

From three patients with laboratory-confirmed influenza A(H1N1)pdm09 virus infection, viral RNA was extracted from throat swabs using the High Pure RNA isolation kit (Roche) with an on-column DNase treatment, according to the manufacturer's protocol. The clinical samples were obtained as part of routine diagnostic procedures in the Academic Medical Centre (Amsterdam, The Netherlands) in accordance with national and institutional regulations concerning the procurement and usage of patient-derived materials.

### Full influenza genome RT-PCR amplification

RT-PCR amplification was performed as described previously (Jonges et al., 2014). In short, for each sample, two separate RT-PCR reactions were performed using primers common-uni12R (5′-GCCGGAGCTCTGCAGATATCAGCRAAAGCAGG-3′), common-uni12G (5′-GCCGGAGCTCTGCAGATATCAGCGAAAGCAGG-3′), and common-uni13 (5′-CAGGAAACAGCTATGACAGTAGAAACAAGG-3′). The first RT-PCR contained the primers common-uni12R and common-uni13. The second RT-PCR contained the primers common-uni12G and common-uni13 that greatly improved the amplification of the PB2, PB1, and PA segments. Reactions were performed using the One-Step RT-PCR kit High Fidelity (Invitrogen) in a volume of 50 μl, and contained 5.0 μl eluted RNA, and final concentrations of 1X SuperScript™ III One-Step RT-PCR reaction buffer, 0.2 μM of each primer and 1.0 μL SuperScript™ III RT/PlatinumTM Taq High Fidelity Enzyme Mix (Invitrogen). Thermal cycling conditions were: reverse transcription at 42°C for 15 min, 55°C for 15 min, 60°C for 5 min; initial denaturation/enzyme activation of 94°C for 2 min; five cycles of 94°C for 30 s, 45°C for 30 s, slow ramp (0.5°C/s) to 68°C, 68°C for 3 min; 30 cycles 94°C for 30 s, 57°C for 30 s, 68°C for 3 min; and final extension of 68°C for 5 min. After the RT-PCR equal volumes of both reactions were combined to produce a well distributed mixture of all eight influenza segments.

### Illumina HiSeq2000 sequencing

Each sample was diluted to a DNA concentration of 50 ng/μL and sheared by nebulization. Samples were then subjected to end-repair, A-overhang, and adaptor ligation with MID-tags using the Illumina TruSeq DNA sample preparation kit. The libraries were multiplexed, clustered, and sequenced on an Illumina HiSeq2000 (TruSeq v3 chemistry) with a paired-end 100 cycles sequencing protocol with indexing according to manufacturer's protocol (Watson et al., 2013; Jonges et al., 2014). After analysis with the Illumina CASAVA pipeline version 1.8.2, resulting datafiles were split into separate sample specific fastq files based on the used multiplex identifier (MID) sequence using the readset parser function of QUASR version 7.0.1 (Cock et al., 2010; Watson et al., 2013).

### Pre-mapping quality control

Quality control (QC) consisted of several bioinformatic steps which were divided in pre-mapping and post-mapping QC. All steps were performed using in-house generated “PHP: Hypertext Preprocessor” (PHP) scripts which are available upon request. Pre-mapping QC steps included the removal of reads from datasets containing uncalled bases (N's) followed by the removal of read parts that exactly matched any of the primers used. Next, the removal of low quality nucleotides from read ends (defined as nucleotides with phred score <30) and removal of low quality read pairs using the paired end QC function from QUASR version 7.0.1 with median phred cutoff of 30 and minimal resulting read length of 50 nucleotides (settings -m30 and -l50) (Watson et al., 2013). Phred quality scores are logarithmically linked to the base call accuracy and a phred score of 30 resembles a base call accuracy of 99.9% while a phred score of 40 represent an accuracy of 99.99% (Ewing and Green, 1998). The selection of a median phred value of 30 as a cutoff value was based on the observation that the largest fraction of reads was maintained in the datasets while still providing a strong reduction in low quality sequence reads (Supplementary Figure 1).

### Read mapping

Datasets from the plasmid pool and both RT-PCR amplified rgWSN33 samples were mapped to the influenza A/WSN/1933(H1N1) reference sequence (taxonomy ID: 382835) using the program Burrows-Wheeler Aligner (BWA) version 0.5.9-r16 with the paired-end mapping option (sampe) and default settings (Li and Durbin, 2009). The three influenza A(H1N1)pdm09 clinical samples were mapped to the A/California/04/2009 H1N1pdm09 virus (taxonomy ID: 641501; Genbank accession numbers FJ966079-FJ966086).

### Post-mapping quality control

Post-mapping QC consisted of the removal of unmapped and unpaired reads from the Sequence Alignment Map (SAM) files as well as removal of reads containing clipped ends (i.e., reads with read sequence parts automatically removed by BWA) and/or insertions/deletions (indels) as identified in the read CIGAR string (Li et al., 2009). Lastly, orphan reads (i.e., read sequences present without a mate) were removed to generate the final cleaned datasets.

### Mismatch frequency (MMF) per sequence read position

For each mapped sequence read, the read sequence, most left-based starting position and mapping information summarized in the CIGAR string were extracted from the SAM file and nucleotides at each mapped read position were compared to the corresponding reference sequence position (Li et al., 2009). The MMF per sequence read position was defined as the percentage of all sequenced nucleotides not matching the reference nucleotide at that read position.

### Influenza genome coverage and MMF per reference sequence position

The coverage per influenza genome position was defined as the total of all mapped nucleotides per reference sequence position with the consensus nucleotide being the mapped nucleotide with the highest frequency. The MMF per reference position was defined as the percentage of all mapped nucleotides excluding the consensus nucleotide frequency.

### Sequence specific error (SSE) analysis

Artificial variation which is introduced by the sequencing process will lead to differences in the MMF in a particular sequencing orientation. Therefore, the MMF at each reference sequence position was determined for reads mapping to the forward and the reverse strand of the reference sequence based on the bitwise flag value (Li et al., 2009). An SSE was defined as a position in which the difference in MMF of nucleotides mapping to the forward and/or reverse strand exceeded the average MMF of all mapped nucleotides at that position, with a minimal sequencing depth of 50 for both mapping orientations.

### Non-uniform spread of mismatches (HCPP analysis) and frequency recalculation

**Figure 1:**
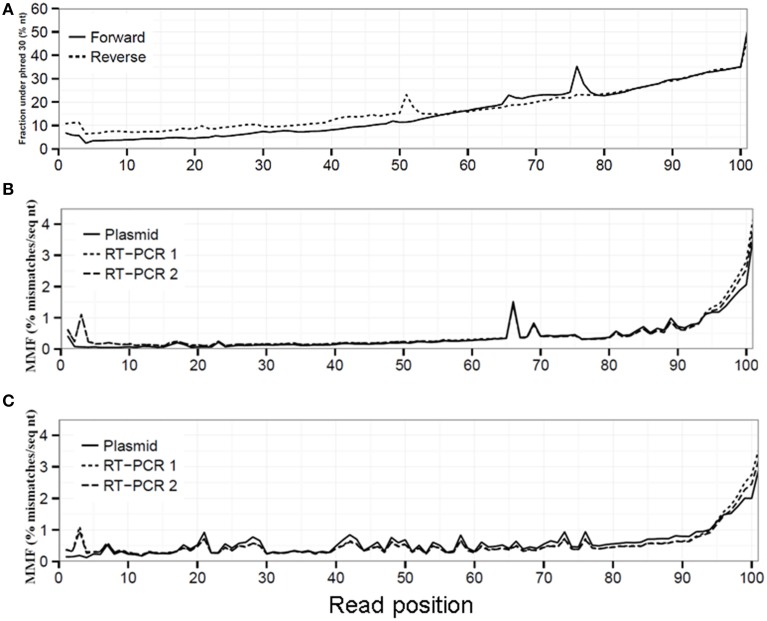
Sequencing quality decreased toward the 3′ read end displayed by an increased fraction of sequenced nucleotides with a phred ≤30 **(A)**. This reduced sequencing quality led to an increased MMF in both the forward **(B)** and reverse **(C)** run for the plasmid, RT-PCR 1 and RT-PCR 2 datasets.

Biological variation will be present in the viral quasispecies in multiple different RNA templates which are separately amplified by RT-PCR, hence DNA fragmentation during library preparation will lead to an even distribution of mutations over the length of the sequence reads (Figure 1A). In contrast, artificial variation by for instance either residual primers/adapters or overamplification of a (subset) of libraries during library amplification will lead to mismatches overrepresented at particular sequence read positions producing a spike-like pattern when visualized in a “hairy caterpillar plot” (HCPP, Figure 1B). Therefore, reads covering influenza genome positions with an MMF above 0.5% were extracted from the cleaned SAM file and the position of each mutation within the read was determined. Artificial variation was arbitrarily defined as a minimal three-fold higher number of mutations at a particular read position above expected and MMF's were recalculated by excluding the excess of mutations.

## Results

### Mismatches introduced by illumina HiSeq2000 sequencing and RT-PCR

Illumina HiSeq2000 sequencing generated 14.2 million read pairs for the reverse genetic plasmid pool sample, and 7.5 million and 4.0 million read pairs for RT-PCR samples 1 and 2, respectively (Table 1). All three samples were sequenced in the same run, hence variation in the plasmid pool sample was assumed to be due to the sample preparation and sequencing process and differences in variation between both RT-PCR samples and the plasmid pool sample were assumed to be due to the RT-PCR amplification step.

**Table 1:** **Overview sequence data**.

		**Plasmid**	**RT-PCR 1**	**RT-PCR 2**	**CS1**	**CS2**	**CS3**
RT-PCR amplified		No	Yes	Yes	Yes	Yes	Yes
Number of read pairs		14,192,571	7,455,869	4,040,876	3,438,654	3,577,913	5,480,898
Number of sequenced nucleotides		2,866,899,342	1,506,085,538	816,256,952	687,730,800	715,582,600	1,096,179,600
Read pairs removed containing N's (% of total)	Forward	0.3	0.3	0.3	0.1	0.1	0.1
	Reverse	0.8	0.8	0.8	0.2	0.2	0.3
Nucleotides removed due to primer/adapter removal (% of total)	Forward	1.4	7.2	7.2	9.2	9.3	9.3
	Reverse	1.2	7.2	7.1	4.2	4.2	11.4
Removed due to median phred read quality <30 (% of total)	Read pairs	6.0	4.4	4.3	4.9	4.5	1.4
	Nucleotides	6.0	4.8	4.7	5.4	5.2	2.4
Total read pairs removed after pre-mapping QC		1,001,818 (7.1%)	399,154 (5.4%)	216,962 (5.4%)	166,215 (5.4%)	159,772 (5.0%)	75,711 (1.8%)
Total nucleotides removed after pre-mapping QC		336,369,593 (11.7%)	228,338,456 (15.2%)	123,364,266 (15.1%)	96,126,836 (14.0%)	98,606,211 (13.8%)	158,272,488 (14.4%)

Read sequence quality is known to decrease toward the 3′ read end (Minoche et al., 2011), which was also observed in our datasets demonstrated by the up to seven-fold increase in the fraction of sequenced nucleotides with a phred value ≤30 in both the forward (from 6.8% at read position 1 to 50.5% at read position 101) and reverse run (from 10.8 to 47.3%, Figure 1A). Without any QC on the sequence reads, this resulted in an average MMF of 0.49% per read position which increased up to 1.58% in the last 10 read positions for both the forward and reverse run (Figures 1B,C, Supplementary Table 1). This increase was only partially due to the before mentioned reduction in sequencing quality, as after applying a phred cutoff of 30 the MMF in the last 10 positions only decreased to 0.36% (Supplementary Table 1). Further investigation of the 3′ sequence read ends with more than two mismatches showed the presence of partial and/or mutated Illumina adapters (data not shown). Furthermore, while the average MMF per read position for both RT-PCR samples were not markedly different when compared to the plasmid pool sample the average MMF in the first 10 nucleotides of the forward run of both RT-PCR samples were increased nearly three-fold compared to the plasmid pool sample (Figures 1B,C). Further investigation showed that this was due to presence of RT-PCR primer sequences (data not shown). If not removed, these Illumina adapter and primer read sequences would lead to erroneous interpretation of biological sequence diversity as these sequences were successfully mapped to the reference sequence.

**Figure 2:**
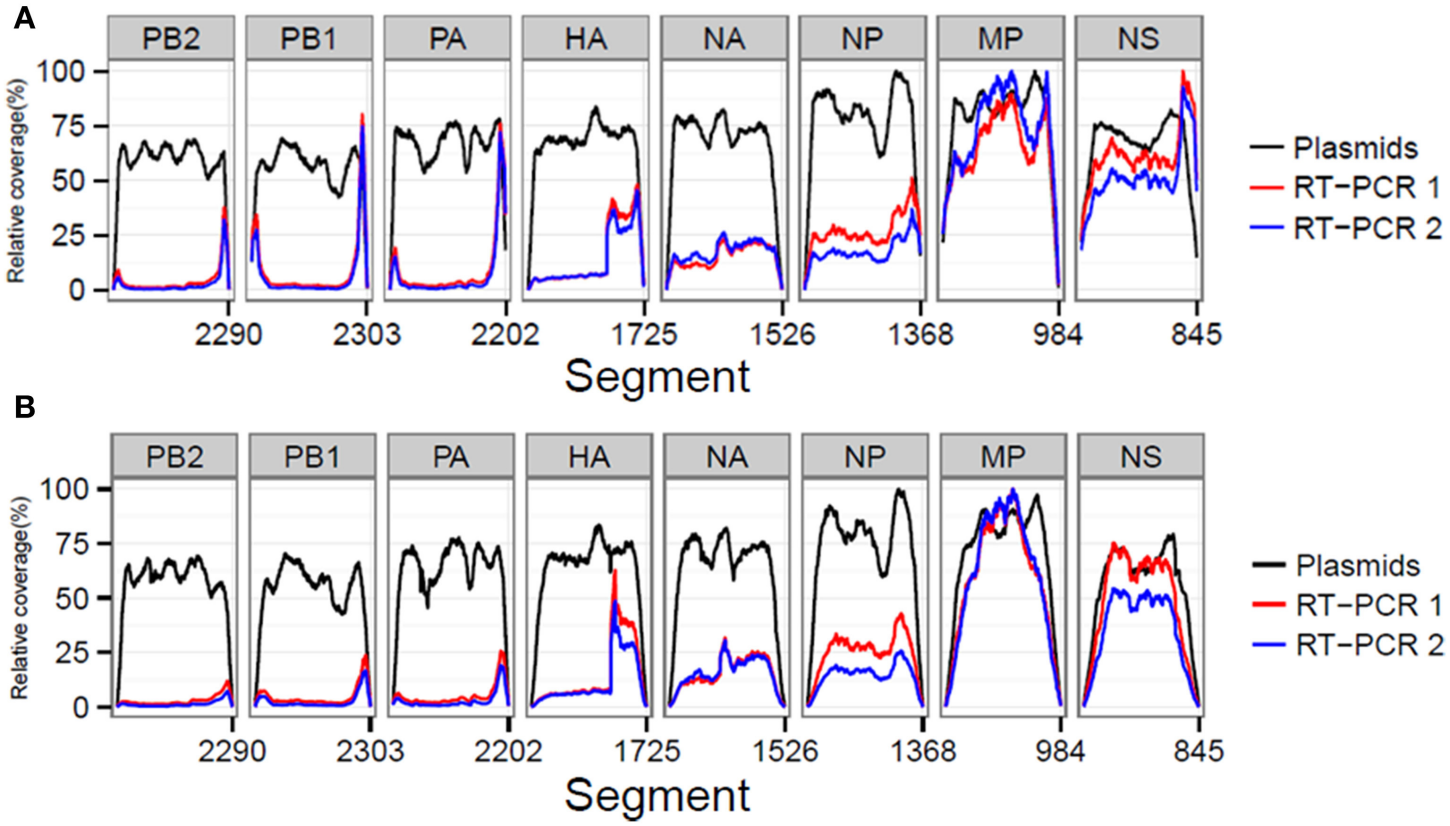
The sequencing process and quality control steps do not lead to a selective bias for particular influenza segments as comparable relative coverages for the plasmid dataset before **(A)** and after **(B)** QC were obtained. In both RT-PCR samples 1 and 2 preferential amplification of the smaller segments was observed.

We also investigated whether the library preparation, sequencing process and RT-PCR amplification influenced the resulting coverage at specific influenza genome positions. In order to compare between samples, the coverage was expressed as a percentage of the maximum obtained coverage within that sample (relative coverage). Results showed that in the plasmid pool sample similar relative coverages were obtained for the different influenza segments indicating that the library preparation and sequencing steps did not create a bias (Figures 2A,B). However, for the RT-PCR amplified samples, we observed increased coverages per position for the smaller influenza segments (Figures 2A,B). In addition, both RT-PCR amplified samples displayed a marked four-fold increase in coverage in the HA segment starting at reference position 1189. At reference position 1176, a Uni13-like primer sequence was identified with only two mismatches compared to the Uni13 primer used for amplification, highly suggestive for non-specific primer binding and subsequent amplification.

In summary, preferential amplification of the smaller segments in the RT-PCR combined with the presence of primer and Illumina adapter sequences led to increased mismatch frequencies per sequence read position, which could impact the accurate detection and identification of viral minority variants.

### Quality control of sequence reads

**Figure 3:**
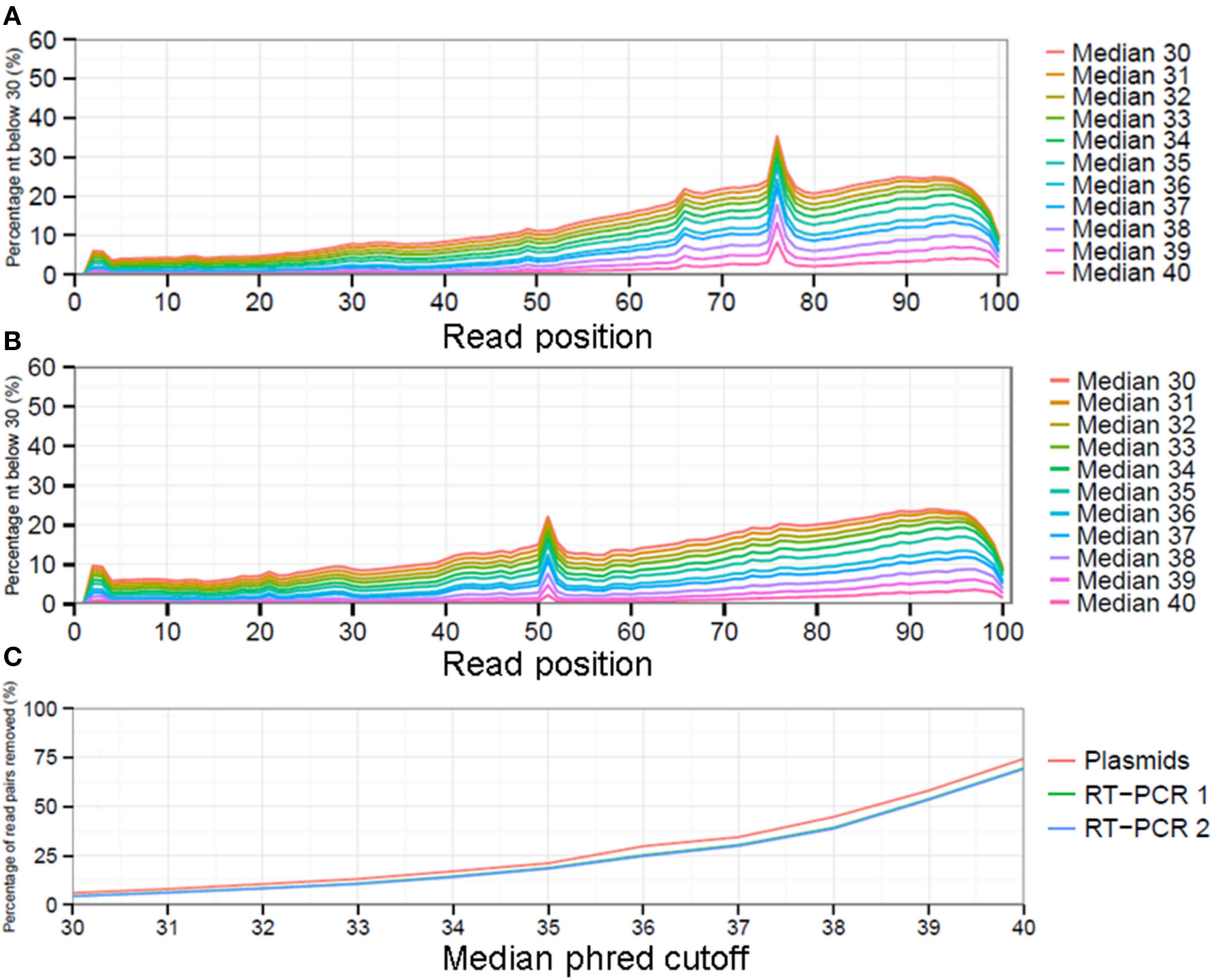
The impact of selecting different median phred quality cutoff values on the remaining reads as displayed by fraction of sequenced nucleotides with a phred ≤30 for the forward **(A)** and reverse **(B)** run. Increasing the minimal median phred read quality will result in increased numbers of read pairs being removed from the analysis **(C)**.

While there is no gold standard for QC, it is generally divided in pre-mapping QC and post-mapping QC steps (see Materials and Methods). In pre-mapping QC read pairs were removed containing uncalled bases, primer sequences as well as low quality nucleotides from read ends. In the final pre-mapping QC step all remaining read pairs were required to have a minimal length of 50 and minimal median phred value of 30. While the selection of a particular median phred cutoff is rather arbitrary, increasing the median phred cutoff value removed increasing numbers of read pairs from the datasets to nearly up to 75% for the plasmid pool dataset with a median phred cutoff of 40 (Figure 3C). With a median phred cutoff of 30, pre-mapping QC led to a total removal of 7.1% of read pairs (11.7% of sequenced nucleotides) from the plasmid pool dataset and 5.4% of read pairs (15.1-15.2% of sequenced nucleotides) from the RT-PCR datasets (Table 1). This lead to a decrease in the fraction of nucleotides with a phred <30 in both the forward and reverse sequence run (Figures 3A,B). The increased percentage of removed nucleotides in the RT-PCR samples was due to removal of primer sequences from the sequence reads.

Subsequently, datasets were mapped to influenza A/WSN/1933(H1N1) consensus sequence and 93.7% of the reads that passed pre-mapping QC were successfully mapped for the plasmid pool dataset, while this was 70.5-70.8% for the RT-PCR datasets, indicating the presence of sequence reads which differed from the used reference sequence (Table 2).

**Table 2:** **Overview of mapping data**.

**Post mapping QC**	**Plasmid**	**RT-PCR 1**	**RT-PCR 2**	**CS1**	**CS2**	**CS3**
Total sequenced reads	28,385,142	14,911,738	8,081,752	6,877,308	7,155,826	10,961,796
Reads passing pre-mapping QC (% of total)	26,381,506 (92.9)	14,113,430 (94.6)	7,647,828 (94.6)	6,504,799 (94.6)	6,795,790 (95.0)	10,766,776 (98.2)
Mapped reads (% of pre-mapping QC passed reads)	24,710,168 (93.7)	9,986,294 (70.8)	5,389,605 (70.5)	6,452,417 (99.2)	6,603,449 (97.2)	10,109,171 (93.9)
Unmapped reads (% of pre-mapping QC passed reads)	1,671,338 (6.3)	4,127,136 (29.2)	2,258,223 (29.5)	52,382 (0.8)	192,341 (2.8)	657,605 (6.1)
Unpaired reads (% of mapped)	982,417 (4.0)	1,168,572 (11.7)	606,869 (11.3)	526,237 (8.2)	513,868 (7.6)	491,450 (4.9)
Clipped reads (% of mapped)	498,217 (2.1)	825,102 (9.4)	420,532 (8.8)	61,272 (0.9)	53,788 (0.8)	936,993 (9.3)
Orphan reads (% of mapped)	649,810 (2.8)	1,523,536 (19.1)	837,872 (19.2)	64,346 (1.0)	56,685 (0.9)	939,518 (9.3)
Total sequenced reads removed by pre- and post-mapping QC (% of total)	5,805,418 (20.5)	8,442,654 (56.6)	4,557,420 (56.4)	1,076,746 (15.7)	1,176,718 (16.4)	3,220,586 (29.4)
Total sequenced nucleotides removed by pre- and post-mapping QC (% of total)	683,255,156 (23.8)	881,452,097 (58.5)	475,561,924 (58.3)	166,794,256 (24.0)	179,258,081 (24.8)	424,048,489 (28.3)

Post-mapping QC steps showed that in RT-PCR datasets 1 and 2, clipped reads (i.e., reads with parts of the read sequence automatically removed by the mapping software) and unpaired reads were nearly four- and three-fold more frequent compared to the plasmid pool dataset respectively. In more detail, these reads predominantly mapped to segment ends and consisted mainly of residual (mutated) primer and adapters sequences (data not shown). Both clipped and unpaired reads were removed from the datasets as well as their mate reads (orphan reads) during further analysis. The percentages of removed reads in the combined QC steps were 20.5% for the plasmid pool dataset and 56.6-56.4% for RT-PCR 1 and RT-PCR 2 respectively, corresponding to 23.8, 58.5, and 58.3% of all sequenced nucleotides respectively (Table 2).

The average MMF per sequence read position was recalculated using the QC-controlled datasets and in the plasmid pool datasets the 20.5% removal of sequence reads led to a 19-fold reduction to an average of 0.02 and 0.03% for the forward and reverse run respectively. Within the RT-PCR amplified samples an average removal of 56.5% of reads only led to a nine-fold reduction to an average of 0.06% (0.05-0.07%, Supplementary Table 1). These results indicate, that not only RT-PCR amplification greatly increased the sample diversity by primarily the introduction of different (mutated) primer sequences, but also that this artificial variation is not adequately removed using current “best practice” QC steps.

### Analysis of unmapped reads for false negative detection of minority variants

While concerns mainly exist for the presence erroneous minority variants, overly stringent QC and removal of reads could also lead to a false negative detection of minority variants. Therefore, sequence reads that passed pre- and post-mapping QC steps but were not mapped to the reference sequence were analyzed for the presence of influenza-like sequences. As unmapped reads were most often unpaired, the reads from the forward and reverse run were analyzed separately. For the plasmid pool sample 17.9 and 15.2% of unmapped reads from the forward and reverse run mapped to reference sequence *Escherichia coli* strain K-12 (Table 3). The source of the *E. coli* was most likely the plasmid isolation, as the plasmids were propagated in *E. coli*. In the RT-PCR samples only a very low percentage of unmapped reads mapped to *E. coli* (0.002 and 0.001%). Human DNA was detected at an average frequency of 1.5% (1.3-1.6%) in the plasmid pool sample while this was only 0.2% (0.1-0.2%) for the RT-PCR samples (Table 3). As the majority of unmapped reads remained of unknown origin, all remaining sequence reads were split in half and both the first and last half of each sequence read were separately mapped to the influenza A/WSN/1933(H1N1) reference sequence. Of the first half of the reads nearly 50% (48.2-50.5%) of the plasmid sample and 57% (55.8-58.0%) of the RT-PCR samples were successfully mapped. For the last half of the sequence read, these percentages were 26% (25.8 and 27.3%) and 37% (35.5-37.9%). Of these mapped reads, nearly 48.8% in the plasmid sample and 0.002% in the RT-PCR samples mapped to the plasmid pHW2000 backbone sequence, while the rest of the mapped read parts were spread over the influenza segment ends. This indicated that the unmapped reads mainly represented the sections where the influenza sequence connected with the plasmid sequence. Interestingly, in 1.0% of split reads, both read halves mapped to the same segment but at opposite ends, while in only 0.03% of split reads the read parts mapped to separate gene segments. This indicates that the ligation step during library preparation is not prone to inducing ligation artifacts. In all three datasets, the reads halves that successfully mapped were predominantly located at the segment ends, indicating that these initially unmappable reads consisted of a combination of a mutated Uni12/Uni13 primer and influenza sequence. In conclusion, these observations suggest that false negatives may occur due to unsuccessful mapping, but the frequency is very likely to be low and mainly restricted to the segment ends.

**Table 3:** **Analysis of the unmapped reads**.

**Post mapping QC**			**Plasmid**	**RT-PCR 1**	**RT-PCR 2**	**CS1**	**CS2**	**CS3**
Total unmapped reads			1,671,338	4,127,136	2,258,223	52,382	192,341	657,605
	Forward run		789,931	1,972,388	1,079,863	26,481	97,125	332,493
	Reverse run		881,407	2,154,748	1,178,360	25,901	95,216	325,112
Unmapped reads mapped to *E. coli*	Forward (%)		141,391 (17.9)	40 (0.002)	16 (0.001)	0 (0.0)	3 (0.0)	4 (0.0)
	Reverse (%)		134,312 (15.2)	41 (0.002)	11 (0.001)	0 (0.0)	3 (0.0)	3 (0.0)
Remaining unmapped reads mapped to human genome sequence hg19	Forward (%)		12,478 (1.6)	4,378 (0.2)	1,788 (0.2)	7,915 (29.9)	78,479 (80.8)	77,225 (23.8)
	Reverse (%)		11,867 (1.3)	4,012 (0.2)	1,592 (0.1)	7,894 (30.5)	77,976 (81.9)	77,363 (23.3)
Remaining unmapped reads split and remapped to corresponding reference sequence	Forward	Front (%)	399,145 (50.5)	1,143,072 (58.0)	621,760 (57.6)	5,555 (29.9)	4,092 (21.9)	98,904 (39.9)
		Back (%)	236,917 (27.3)	824,898 (37.3)	456,299 (37.9)	8,195 (44.1)	8,690 (46.6)	82,033 (33.1)
		Plasmid (%)	48.8	0.002	0.002	NA	NA	NA
	Reverse	Front (%)	424,536 (48.2)	1,202,129 (55.8)	667,583 (56.7)	4892 (27.2)	3954 (22.9)	106,518 (41.8)
		Back (%)	310,692 (25.8)	948,566 (35.8)	509,174 (35.5)	6713 (37.3)	6305 (36.6)	85,395 (33.5)
		Plasmid (%)	49.0	0.002	0.001	NA	NA	NA

*NA, not available*.

### Detection of viral minority variants

Pre- and post-mapping QC steps reduced the average MMF per reference genome position in both RT-PCR samples to 0.08 and 0.09% respectively and to 0.03% in the plasmid pool sample, with only minor differences between the individual influenza segments (Supplementary Table 2). In further detail, the plasmid pool sample had only one position with a MMF greater than 0.5%, while RT-PCR sample 1 had 84 positions and RT-PCR sample 2 had 139 positions above this percentage. Minority variants at these positions would normally be classified as “true” biological minority variants while a fraction actually reflects artificially introduced variation.

### Sequence specific error (SSE) analysis and detection of artificial minority variants

**Figure 4:**
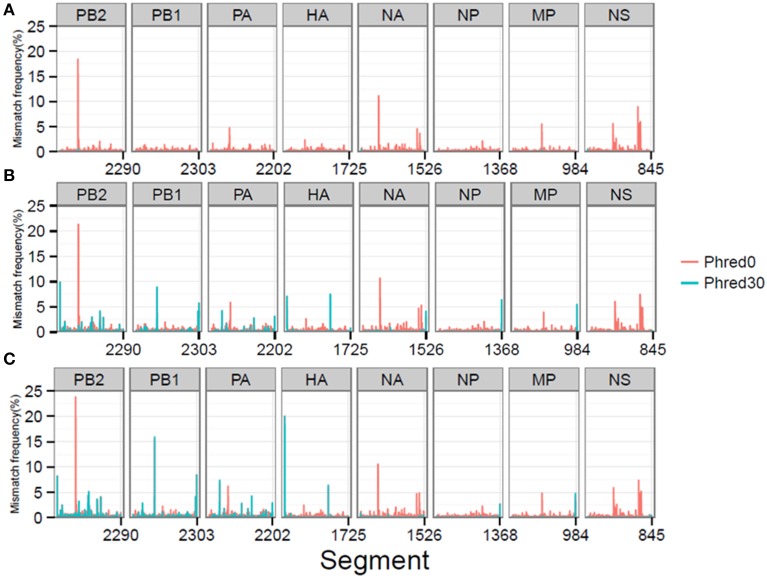
MMF's differ at specific reference genome positions depending on the per nucleotide phred quality cutoff for the plasmid pool **(A)**, RT-PCR 1 **(B)**, and RT-PCR 2 **(C)** samples.

**Figure 5:**
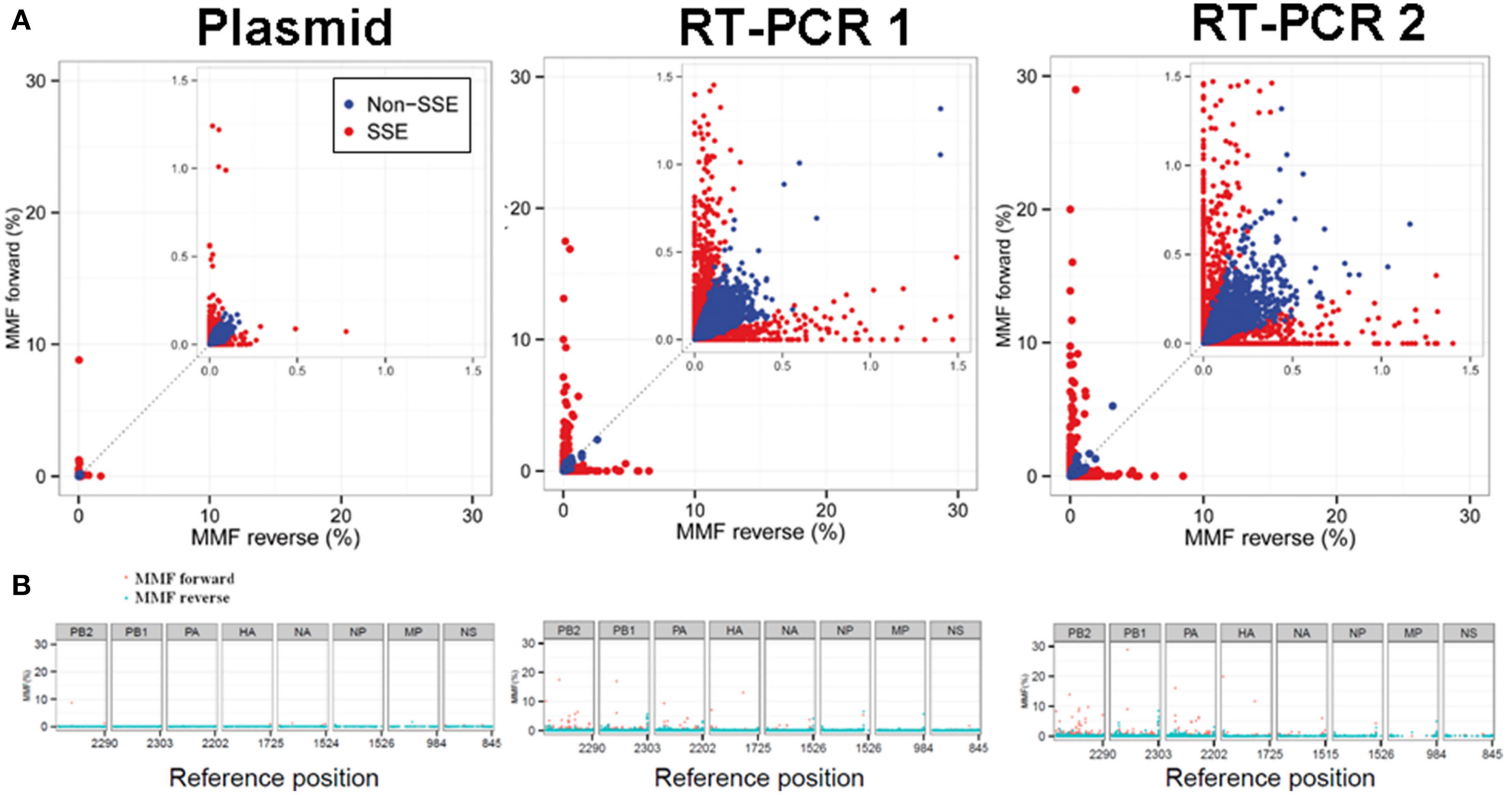
Sequence specific error (SSE) was defined as a position at which the difference in MMF of nucleotides mapping to the forward and the reverse strand exceeded the average MMF. Positions that were classified as SSE are highlighted in red. Both RT-PCR samples showed increased MMF's compared to the plasmid sample indicating introduction of mismatches due to the RT-PCR amplification process **(A)**. The distribution of SSE's over the influenza genome was primarily located in the large polymerase segments of the RT-PCR samples and at segment ends **(B)**.

At a combined total of 224 positions, in three separate samples, the MMF was above 0.5%. These positions were located at specific sites in the influenza genome (Figure 4). For instance, in all three samples an increased MMF was detected at reference position 666 of PB2. Without a per nucleotide quality cutoff, the MMF's at this position were 18.4% in the plasmid sample and 21.3-23.8% in the RT-PCR samples, while these were 0.6%, and 1.1-1.3% after applying a phred quality cutoff of 30. What was striking was that the mismatches at this position were unevenly distributed between the reads mapping to the forward and reverse strand. In the plasmid sample, the MMF was 8.8% in reads mapping to the forward strand and 0.04% in reads mapping to the reverse strand, indicating a strand bias. The specific T666G mutation was located in a homopolymer region and it has been known that the Illumina platform has increased error rates at homopolymeric regions (Minoche et al., 2011). After SSE analysis (Figure 5) and removal of positions at which the MMF was associated with a strand bias, no positions with an MMF above 0.5% remained in the plasmid sample while in RT-PCR samples 1 and 2 only 6 (out of 84) and 21 (out of 139) positions remained. Interestingly, 5 of the 6 positions in RT-PCR 1 were also identified in RT-PCR 2, with only 0.1-0.3% difference in mismatch frequencies, indicating that these minority variants were either present prior to RT-PCR (i.e., introduced by the reverse genetics procedure) or that these particular errors were specifically introduced by the RT-PCR step.

### Detection of artificial variation due to non-random distribution of mismatches

**Figure 6:**
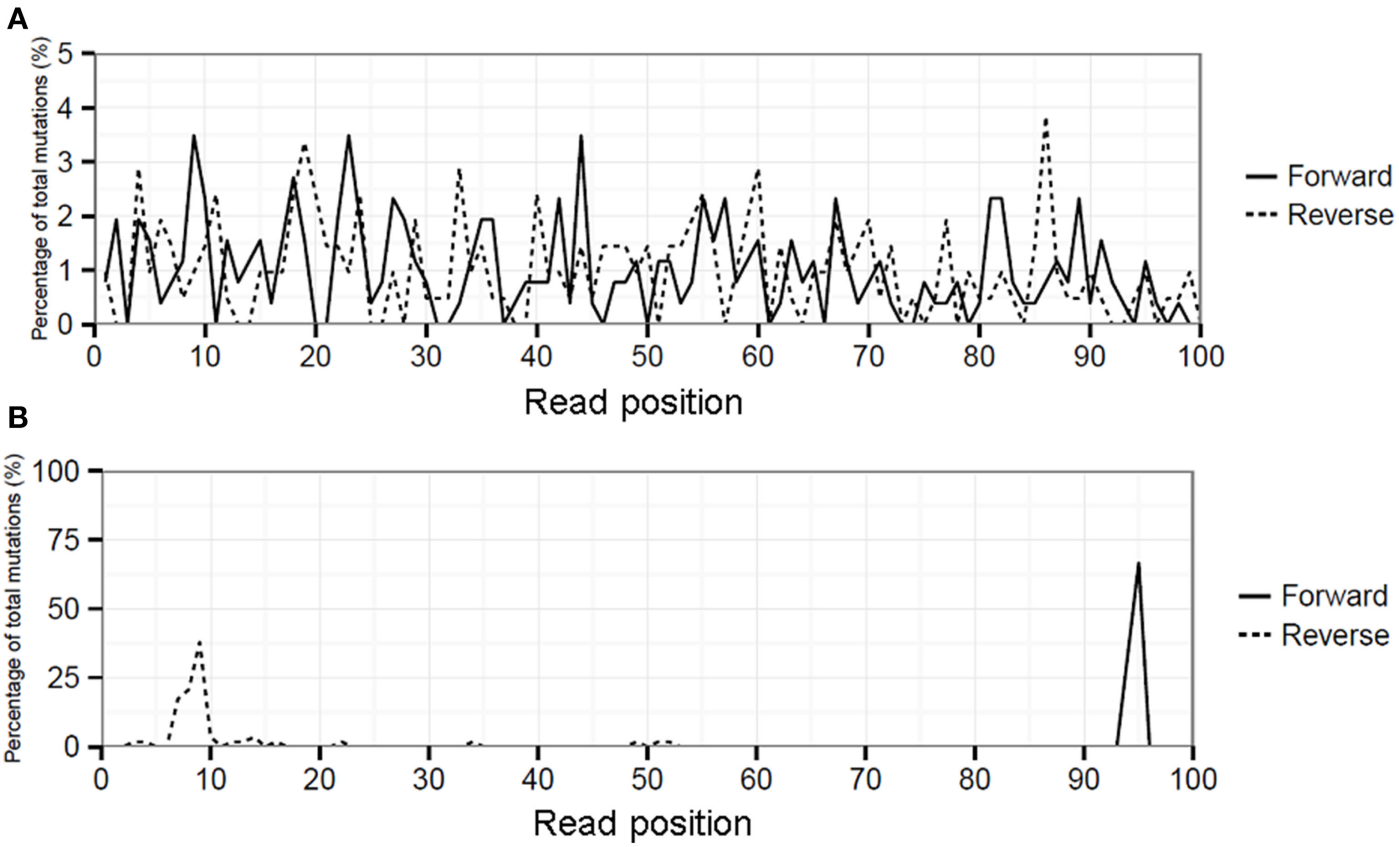
Hairy caterpillar plot (HCPP) of RT-PCR 1 influenza position PA-1976 **(A)** which showed that mismatches at this position were spread evenly over all read positions and strand orientations, indicative for an actual biological variation. At position PA-2189 **(B)** the mismatches were predominantly present at specific read positions in both strand orientations indicative for artificial variation.

Artificial variation can be identified by the non-random distribution of mismatches over the sequence reads using hairy caterpillar plots (HCPP analysis). For instance, influenza position 1976 of PA had a MMF in RT-PCR samples 1 and 2 of 1.2 and 1.5%, respectively. HCPP analysis showed an even spread of the mismatches over the length of the sequence reads (Figure 6A). This even dispersal of mismatches indicated that the increased MMF was not related to any technical error and the corrected mismatch frequencies were therefore nearly identical at 1.1 and 1.0%. In contrast, influenza position 2189 of PA showed mismatch frequencies of 0.70 and 0.85% in RT-PCR samples 1 and 2 and HCPP analysis showed that the mutations were primarily located within the first and last 10 positions of the reads mapping to the forward and reverse strand respectively (Figure 6B). This indicated that variation is introduced artificially by for instance (over)amplification of the mutation in the library preparation. By the removal of excess mutations the corrected mismatch frequencies decreased for both RT-PCR samples to 0.2%.

After the HCPP analysis, two of the six positions in RT-PCR sample 1 remained with a MMF above 0.5% and 12 of 21 positions in RT-PCR sample 2. Both remaining positions from RT-PCR sample 1 were also identified in RT-PCR sample 2 (positions PA-1976 and PB1-1935) with similar mismatch frequencies. As the HCPP analysis is dependent upon adequate genome coverage it may suggest that reduced coverage is the primary cause of failing to detect the remaining artificially introduced variation. Indeed, the average coverage of the 10 remaining positions was 1.489 (675–3.382), which was nearly 22-fold lower compared the average genome coverage of 33.086 for RT-PCR sample 2.

In conclusion, with the addition of the SSE and HCPP analysis the positions with increased MMF decreased from 86 to 2 positions in RT-PCR 1 and from 139 to 12 positions in RT-PCR 2 corresponding to an average reduction of 95% (91.4-97.7%). In addition, the positions with the highest initial mismatch frequencies in RT-PCR 1 and 2 were reduced from an initial 8.9 and 15.9% to 1.1 and 1.5%, respectively. This 1.5% limit reflects the lowest frequency at which artificial variation remained clearly distinguishable from biological variation after all pre- and post-mapping steps as well as SSE and HCPP analysis.

### Applicability to clinical samples

The detection of minority variants in clinical samples could be complicated by additional (unknown) factors, such as low viral load, multiple rounds of viral replication and/or the presence of bacterial, viral or human DNA. Therefore, our approach was applied to three influenza A(H1N1)pdm09 virus positive clinical samples, which generated between 3.4 and 5.5 million read pairs for each clinical sample (Table 1). Less than 5.0% of the read pairs of each dataset were removed in pre-mapping QC steps. The number of unmapped reads per sample were 0.8% for clinical sample CS 1, 2.8% for CS2 and 6.1% for CS3 (Table 2) and predominantly consisted of reads which mapped to the human genome with substantial variation between samples (23.3-81.9%). Additional BLAST analysis also showed the presence of reads of bacterial origin in all three clinical samples consistent with the type of clinical specimen used (data not shown). After splitting the remaining unmapped reads in two, influenza was still detected in all three samples (Table 3). As observed with the RT-PCR datasets, read parts were detected that mapped to opposite ends of gene segments particularly in the large polymerase genes (PB2, PB1, and PA) in all three clinical samples. After pre- and post-mapping QC steps, 15.7, 16.4, and 29.4% of sequenced reads were removed from CS1, CS2, and CS3 datasets, respectively (Table 2).

The average mismatch frequencies per influenza genome position were calculated and a total of 28, 72, and 162 positions with a MMF above 0.5% were identified in CS1, CS2, and CS3, respectively. The highest mismatch frequencies were found in HA for CS1 and CS2 [reference position 590 for CS 1 (12.5%) and reference position 261 for CS2 (10.7%)] while for CS3 the highest MMF was found in two adjacent positions in PB2 [position 275 and 276 (25.0 and 11.9%)]. After SSE analysis, 27 positions remained in CS1, 70 positions in CS2 and 86 positions in CS3. HCPP analysis further reduced the number of positions to 16 in CS 1, 58 positions in CS2 and 66 positions in CS3. As the lowest mismatch percentage at which biological variation was distinguishable from artificial variation was 1.5% this frequency was used as a cutoff value and five positions remained in CS1, 23 in CS2 and 5 in CS3. At these positions, most nucleotide mutations in CS1 and CS3 were silent (four out of five for both samples), 14 out of 23 nucleotide mutations in CS2 lead to amino acid substitutions of which the highest frequencies were observed in the HA gene (Table 4). A BLAST search for the HA-N81D substitution showed that this variant has been identified in Japanese patients. Similarly, several other HA substitutions were also identified in patients, indicating that these variants are likely to be actual biological variants.

**Table 4:** **Minority amino acid variants above 1.5% in clinical samples after QC and SSE/HCPP analysis**.

**Sample**	**Identified minority variants (>1.5%)**	
	**Number of synonimous mutations**	**Identified non-synonimous mutations (%)**
CS1	4	PA E300K (3.1%)
CS2	9	HA N81D (10.5%), HA L547V (2.5%), PA I438L (2.3%), NP D151N (2.2%), HA S123G (2.1%), PB2 D153N (2.0%), PB2 P200H (2.0%), NP A342T (2.0%), NP A33T (1.9%), NA P328H (1.7%), NA I32M (1.7%), PB2 A204E (1.6%), PB1 D638H (1.5%), HA G519E (1.5%)
CS3	4	PB1 T42K = PB1-F2 Q11K (1.7%)

These results showed that a high proportion of observed minority variants were introduced artificially and could be identified and removed efficiently with proper QC steps and additional SSE and HCPP analyses without the need for complicated mathematical haplotype reconstruction algorithms. This ultimately resulted in a reduction of false-positive detection of minority variants hence improving the data resolution and identification of true biological variants. We emphasize that while variation caused by artificial introduction was successfully removed; frequencies of assumed biological minority variants in the clinical samples remained nearly unchanged after the additional SSE and HCPP analysis steps.

## Discussion

In this study, the average MMF per read position related to the Illumina Hiseq2000 sequencing process was 0.5% while in the last 10 read positions the MMF increased to 1.6% primarily due to the presence of (mutated) Illumina adapters. The MMF after RT-PCR amplification and sequencing was determined using RNA from virus produced by reverse genetics using cells which do not support productive viral replication. As the virus progeny will have been transcribed from the same plasmids as used to determine the sequencing-related MMF, sequence variation is assumed to only result from mismatches introduced during this process or the subsequent RT-PCR. In comparison to the plasmid control sample, the MMF in the first 10 read positions increased due to the presence of primer sequences used in RT-PCR amplification, while the overall MMF remained nearly identical. The finding of increased MMF's at read ends are in line with previous observations using the Illumina Genome Analyzer platform in the quasispecies analysis of foot-and-mouth disease (Wright et al., 2011).

After pre- and post-mapping QC steps, the average MMF per read position in the RT-PCR amplified datasets reduced to 0.06% overall and 0.1% in the last 10 read positions. This represents the lowest possible technical MMF, indicating that minority variants with a frequency below 0.1% will remain indistinguishable from technical error. Further increasing the sequencing depth will not improve the identification of these very low frequency (<0.1%) minority variants without additional analysis steps for the detection of artificial variation. In this study, after “best practice” QC, 223 influenza genome positions remained which would normally be considered to be “true” biological variants, while 95% were in fact artifacts, hampering reliable interpretation of the sequence data and hindering efficient follow-up studies. These artifacts were not randomly distributed over the influenza virus genome but were shown to be specifically influenced by the sequence context, detectable by a mutational strand bias. The SSE analysis indicated that nearly 90% of the positions with increased MMF were artifacts. Furthermore, shearing or nebulization of amplified DNA during library preparation will lead to biological variant mutations to be evenly spread over the sequence read. When a particular fragment containing a mutation is preferentially (over)amplified during library preparation (i.e., resequenced due to low copy number) this random spread would be disrupted and the prevalence of this mutation overestimated. This bias has been described before and complicated time-consuming laboratory methods, like primer-ID tagging, have been developed that can identify and correct for this (Jabara et al., 2011). We designed a more straightforward approach, the HCPP analysis, that was able to simultaneously detect variation associated with non-specific RT-PCR products, residual primer and adapter nucleotides and preferential overamplification, as each would lead to disruption of the random spread. The HCPP analysis not only provides qualitative information on the presence of artifacts, but also allows for correction of a specific MMF by subtracting a quantifiable artifact. The HCPP analysis can in theory be applied to all shotgun-based deep sequencing datasets as the principle of artifact identification and quantification is not based on the sequencing platform but on the shearing of the DNA template during library preparation. However, as the cutoff value at which variation is defined as artificial is determined by the division of detected mutations by the sequence read length, it lacks sensitivity when either the sequencing depth or the MMF for the genome position in question are low. A solution to this problem would be to perform replicate RT-PCR reactions.

Combined, the SSE and HCPP analysis led to a near 95% reduction of identified positions with increased MMF's in RT-PCR amplified genomes from reverse-genetics generated virus samples, providing a simple and efficient method to reduce artifacts and more reliable identification of true minority variants. While in clinical samples a larger fraction of the positions with increased mismatch frequencies can be assumed to be due to biological variation, the lowest reduction in positions we obtained was still nearly 68%. The impact of HCPP analysis was greatest in clinical sample CS3, which also had the highest percentage of nucleotides removed during pre-mapping QC steps, particularly the primer/adapter removal. This indicates that most of the initial variation was due to remaining mutated primers which were efficiently recognized and removed during the HCPP analysis step. Our study clearly demonstrates that without proper testing for technical errors, in particular for strand bias, overestimation of minority variants (and their frequencies) in biological samples is very likely to occur.

This is the first study that describes in detail the MMF's of full genome influenza RT-PCR amplification combined with Illumina HiSeq2000 sequencing. We present additional analysis steps to remove artifacts leading to a 95% reduction of otherwise erroneously identified minority variants in plasmid controlled datasets and a 68% reduction in clinical samples. Our approach improved the distinction between artifactual and biological variants in high throughput sequencing datasets, hence facilitating studies requiring reliable high resolution identification of viral minority variants.

## Author contributions

Matthijs R. A. Welkers designed the scripts, performed the bioinformatic analysis and wrote the manuscript. Marcel Jonges helped design the experiments, assisted in data analysis and writing of the manuscript. Rienk E. Jeeninga performed the RT-PCR amplification and influenza reverse genetics experiments. Marion P. G. Koopmans and Menno D. de Jong conceived and supervised the study and contributed to writing of the manuscript. All authors read and approved the final manuscript.

## Funding

This work was supported by funding from the European Community's Seventh Framework Programme [FP7/2007–2013] under the project EMPERIE, EC grant agreement number 223498; from the Dutch Ministry of Economic Affairs, Agriculture, and Innovation, under the Castellum Project; and from the Academic Medical Centre (AMC) Graduate School [PhD scholarship to MRAW].

### Conflict of interest statement

The authors declare that the research was conducted in the absence of any commercial or financial relationships that could be construed as a potential conflict of interest.
