# Supplementary material for: Improved detection of artifactual viral minority variants in high-throughput sequencing data
Source: Front Microbiol. 2015 Jan 22;5:804. doi: 10.3389/fmicb.2014.00804 (PMC4302989; doi:10.3389/fmicb.2014.00804)
Supplement: Supplementary file 1 [file Table1.DOC]

**Supplementary table 1** Average mismatch frequencies (MMF) per read position before and after QC and phred quality cutoff

| **Before QC** |  | **Plasmid** | | **RT-PCR 1** | | **RT-PCR 2** | |
| --- | --- | --- | --- | --- | --- | --- | --- |
|  | Run | *Forward  (% of seq. nt)* | *Reverse  (% of seq. nt)* | *Forward  (% of seq. nt)* | *Reverse  (% of seq. nt)* | *Forward  (% of seq. nt)* | *Reverse  (% of seq. nt)* |
| **No quality control** | *Average* | 0.39 | 0.58 | 0.46 | 0.57 | 0.44 | 0.53 |
|  | *First 10* | 0.09 | 0.24 | 0.32 | 0.43 | 0.32 | 0.40 |
|  | *Last 10* | 1.56 | 1.58 | 1.87 | 1.82 | 1.71 | 1.66 |
|  |  |  |  |  |  |  |  |
| **Phred cutoff 30** | *Average* | 0.06 | 0.09 | 0.14 | 0.17 | 0.13 | 0.15 |
|  | *First 10* | 0.04 | 0.03 | 0.24 | 0.22 | 0.24 | 0.20 |
|  | *Last 10* | 0.36 | 0.39 | 0.72 | 0.81 | 0.66 | 0.72 |
| **After QC** |  |  |  |  |  |  |  |
| **No quality control** | *Average* | 0.16 | 0.23 | 0.19 | 0.25 | 0.18 | 0.24 |
|  | *First 10* | 0.05 | 0.10 | 0.11 | 0.18 | 0.11 | 0.18 |
|  | *Last 10* | 0.28 | 0.30 | 0.24 | 0.26 | 0.23 | 0.25 |
|  |  |  |  |  |  |  |  |
| **Phred cutoff 30** | *Average* | 0.02 | 0.03 | 0.05 | 0.07 | 0.05 | 0.06 |
|  | *First 10* | 0.03 | 0.03 | 0.09 | 0.10 | 0.09 | 0.10 |
|  | *Last 10* | 0.01 | 0.02 | 0.04 | 0.05 | 0.04 | 0.04 |
