# Supplementary material for: Improved detection of artifactual viral minority variants in high-throughput sequencing data
Source: Front Microbiol. 2015 Jan 22;5:804. doi: 10.3389/fmicb.2014.00804 (PMC4302989; doi:10.3389/fmicb.2014.00804)
Supplement: Supplementary file 2 [file Table2.DOC]

**Supplementary table 2.** Average mismatch frequencies per gene segment after applying a phred quality cutoff of 30

| **Segment** | **Plasmid** | **RT-PCR 1** | **RT-PCR 2** | **CS1** | **CS2** | **CS3** |
| --- | --- | --- | --- | --- | --- | --- |
|  |  |  |  |  |  |  |
| PB2 | 0.03 (0 – 0.6) | 0.09 (0 – 4.1) | 0.11 (0 – 5.1) | 0.04 (0 - 1.9) | 0.03 (0 - 2.5) | 0.09 (0 - 25.0) |
| PB1 | 0.03 (0 – 0.2) | 0.10 (0 – 10.0) | 0.12 (0 – 15.9) | 0.04 (0 - 1.2) | 0.03 (0 - 6.7) | 0.06 (0 - 11.8) |
| PA | 0.03 (0 – 0.1) | 0.09 (0 – 4.2) | 0.10 (0 – 7.4) | 0.04 (0 - 3.4) | 0.03 (0 - 2.8) | 0.07 (0 - 4.6) |
| HA | 0.03 (0 – 0.2) | 0.07 (0 – 7.5) | 0.06 (0 – 6.4) | 0.05 (0 - 12.5) | 0.03 (0 - 10.7) | 0.04 (0 - 0.8) |
| NA | 0.03 (0 – 0.6) | 0.07 (0 – 6.5) | 0.06 (0 – 2.8) | 0.04 (0 - 1.0) | 0.03 (0 - 2.1) | 0.03 (0 - 1.2) |
| NP | 0.03 (0 – 0.6) | 0.07 (0 – 4.1) | 0.06 (0 – 0.7) | 0.04 (0 - 0.7) | 0.04 (0 - 2.5) | 0.04 (0 - 1.4) |
| MP | 0.03 (0 – 0.4) | 0.07 (0 – 5.5) | 0.07 (0 – 4.8) | 0.04 (0 - 0.6) | 0.03 (0 - 1.9) | 0.04 (0- 0.5) |
| NS | 0.03 (0 – 0.1) | 0.06 (0 – 0.3) | 0.06 (0 – 0.4) | 0.05 (0 - 4.3) | 0.03 (0 - 1.2) | 0.04 (0 - 0.6) |
|  |  |  |  |  |  |  |
| Average mmf per genome position (%) | 0.03 (0 – 0.6) | 0.08 (0 - 10) | 0.09 (0 - 20) | 0.04 (0 - 12.5) | 0.03 (0 - 10.7) | 0.06 (0 - 25.0) |
