# Supplementary figures and images for: Improved detection of artifactual viral minority variants in high-throughput sequencing data
Source: Front Microbiol. 2015 Jan 22;5:804. doi: 10.3389/fmicb.2014.00804 (PMC4302989; doi:10.3389/fmicb.2014.00804)

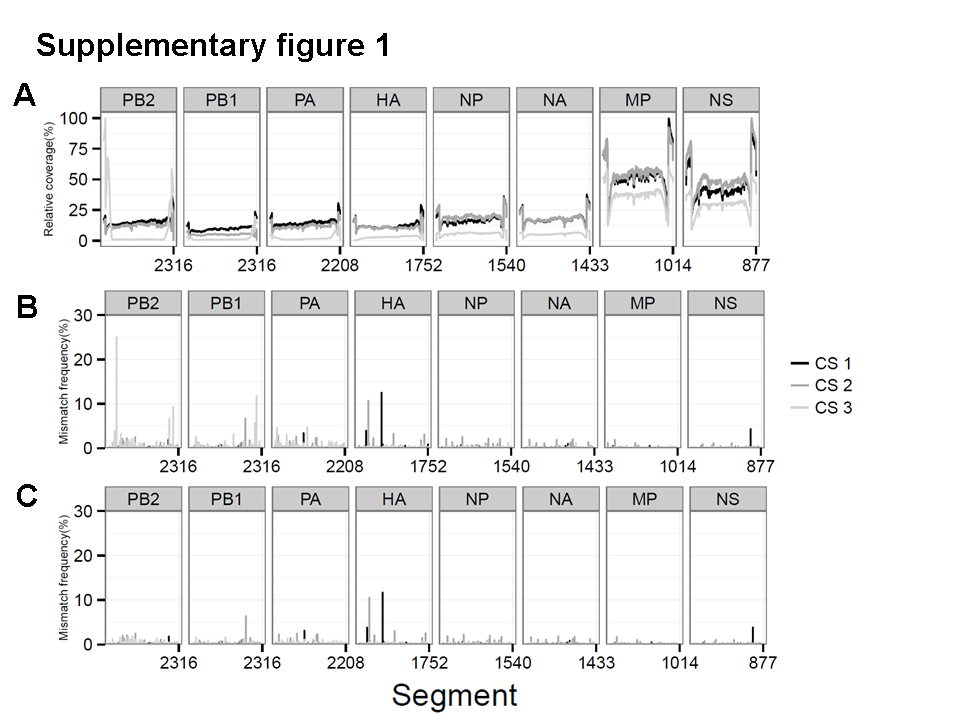

Supplement: Supplementary Figure 1 — Relative coverage per influenza genome position after pre- and post-mapping QC (A) and the MMF's before (B) and after (C) application of the SSE and HCPP analysis for the clinical samples CS1, CS2 and CS3. [file Image1.TIF]
